# Enhanced diffusion of Uranium and Thorium linked to crystal plasticity in zircon

**DOI:** 10.1186/1467-4866-7-10

**Published:** 2006-12-20

**Authors:** Nicholas E Timms, Peter D Kinny, Steven M Reddy

**Affiliations:** 1 Department of Applied Geology, Curtin University of Technology, GPO Box U1987, Perth WA 6845, Australia

## Abstract

The effects of crystal-plasticity on the U-Th-Pb system in zircon is studied by quantitative microstructural and microchemical analysis of a large zircon grain collected from pyroxenite of the Lewisian Complex, Scotland. Electron backscatter diffraction (EBSD) mapping reveals a *c*.18° variation in crystallographic orientation that comprises both a gradual change in orientation and a series of discrete low-angle (<4°) boundaries. These microstructural data are consistent with crystal-plastic deformation of zircon associated with the formation and migration of dislocations. A heterogeneous pattern of dark cathodoluminescence, with the darkest domains coinciding with low-angle boundaries, mimics the deformation microstructure identified by EBSD. Geochemical data collected using the Sensitive High Resolution Ion MicroProbe (SHRIMP) shows a positive correlation between concentrations of the elements U, Th and Pb (ranging from 20–60 ppm, 30–110 ppm, and 14–36 ppm, respectively) and Th/U ratio (1.13 – 1.8) with the deformation microstructure. The highest measured concentrations and Th/U coincide with low-angle boundaries. This enrichment is interpreted to reflect enhanced bulk diffusion of U and Th due to the formation and migration of high-diffusivity dislocations. ^207^Pb/^206^Pb ages for individual analyses show no significant variation across the grain, and define a concordant, combined mean age of 2451 ± 14 Ma. This indicates that the grain was deformed shortly after initial crystallization, most probably during retrograde Inverian metamorphism at amphibolite facies conditions. The elevated Th over U and consistent ^207^Pb/^206^Pb ages indicates that deformation most likely occurred in the presence of a late-stage magmatic fluid that drove an increase in the Th/U during deformation. The relative enrichment of Th over U implies that Th/U ratio may not always be a robust indicator of crystallization environment. This study provides the first evidence of deformation-related modification of the U-Th system in zircon and has fundamental implications for the application and interpretation of zircon trace element data.

## Background

Cathodoluminescence (CL) and backscattered electron (BSE) imaging of zircon (ZrSiO_4_) commonly records fine-scale composition zoning [1] that demonstrates its ability to retain geochemically important trace and rare earth elements (REE) over a range of geological conditions. This attribute has resulted in its widespread application to a variety of Earth Science disciplines [2-8]. Two important element in zircon, U and Th, form tetravalent cations that substitute for Zr^4+ ^[9] at values typically between 5–4000 ppm and 2–2000 ppm respectively, and are useful for two main reasons. Firstly, the Th/U ratio of zircon is characteristic of its crystallization environment, such that Th/U >0.2 are associated with crystallization from a melt while metamorphic zircon records Th/U< 0.07 [2,10]. Secondly, zircon has very low initial amounts of Pb due to the incompatibility of Pb^2+ ^in the zircon lattice. Consequently, the radioactive decay of U and Th to various isotopes of Pb provides a valuable geochronological tool that provides constraints on a range of geological processes, for example the timing of melt crystallization or high temperature metamorphism. However, fundamental to the application of zircon geochemistry to geological studies is knowledge of the transport mechanisms of U, Th and Pb over a range of crustal conditions.

Empirically-derived volume diffusion parameters of U, Th and Pb indicate that these elements are essentially immobile at temperatures below *c*.900° C [9,11-15]. Despite the low diffusivities of U, Th and Pb for volume diffusion, some studies indicate element mobility (particularly Pb loss) at low temperature conditions [16-18]. Intracrystalline damage associated with radiation damage (metamictization), particularly within U-rich zircon, can lead to fast-diffusion pathways which allow U, Th and Pb migration at low temperatures [19,20]. However, it has been demonstrated that Pb mobility may be independent of U and Th concentration, and hence level of radiation damage [21], indicating that other processes must have contributed to elemental migration.

Deformation of zircon at crustal conditions by brittle failure has been demonstrated during mylonitization, or due to volume changes associated with metamictization [22-24]. Plastic microstructures such as planar deformation features have been reported in shocked zircon [25-27]. Quantitative microstructural studies have found that zircon may deform by crystal-plastic processes, manifest by heterogeneously distributed, discrete low-angle boundaries (<0.5 μm wide) that define deformation bands/subgrains with gradually distorted interiors [28,29]. This deformation can have profound effects on intragrain REE distribution (i.e., can enhance REE diffusion distances by at least five orders of magnitude), because the formation and migration of dislocations during plastic deformation provide high diffusivity pathways for element migration [28]. Such findings in zircon are in agreement with other studies which show a defect control on element mobility [30-33]. However, the role of deformation-related microstructures in zircon on the migration of U and Th is currently unknown. This study addresses this specific area and combines quantitative electron backscatter diffraction with cathodoluminescence and ion microprobe analyses to investigate the microstructural control of U-Th compositional variations and migration in zircon from a syn-metamorphic pyroxenite from the Lewisian Gneiss Complex, NW Scotland.

### Sample Geological Framework and Sample Description

**Figure 1:**
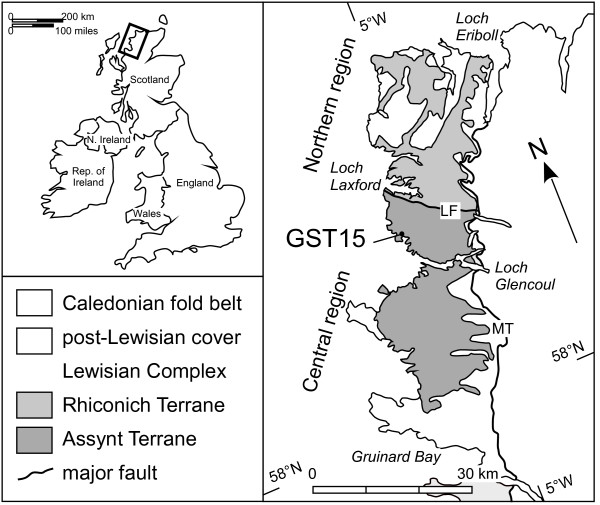
Simplified geological map of NW Scotland showing the location of sample GST15. The Assynt terrane is bounded to the north by the Laxford Front (LF) and to the east by the Moine thrust (MT). Adapted from Kinny and Friend [34].

The sample (GST15 [34]) comprises a large (c. 12.5 mm long), pale brown, single crystal of zircon with an aspect ratio of ~4:1:1 shape, does not preserve well developed crystal faces and has rounded, uneven terminations. It was taken from a cluster of large zircon grains at the pegmatitic margin of a pyroxenite intrusion in the Assynt terrane (central region) of the Archaean Lewisian complex of northwest Scotland (Fig. 1). The Assynt terrane is characterized by 'Badcallian' granulite facies peak metamorphism and associated magmatism at 2,480–2,490 Ma, and localized retrogression and hydrous deformation during subsequent episode(s) of 'Inverian' amphibolite facies metamorphism [34,35]. The region has been further heterogeneously overprinted by amphibolite-grade, Proterozoic Laxfordian metamorphism/deformation. The unaltered host pyroxenite body is one of a series of ultramafic bodies at Loch an Daimh Mór (*ca*. 58° 19'54"N, 5° 07'57"W), north of Upper Badcall, and is considered to have intruded close to or during peak metamorphism and is surrounded by relatively unretrogressed granulite facies gneisses [36,37]. Previous analysis of the grain using SHRIMP has shown that it contains 16–50 ppm U, 15–110 ppm Th, and has produced a concordant, mean ^207^Pb/^206^Pb age of 2470 ± 30 Ma [34]. This grain was chosen because of its old age and complex regional metamorphic/deformation history and so has potential for Pb loss over time, and its large size allows multiple SHRIMP analyses across the grain to facilitate investigation of a wide range of microstructural sites.

### Analytical techniques

The epoxy resin-mounted zircon grain was mechanically polished with diamond paste such that 8 mm of the grain is exposed at the sample surface, and then given a final polish using a colloidal silica suspension in pH 9.5 NaOH solution on a Buehler Vibromet II™ polisher for 4 hours to remove the surface damage from mechanical polishing. Crystallographic orientation information was collected via automated electron backscatter diffraction (EBSD) mapping using facilities at Curtin University of Technology, Perth. Details of the system and settings are given in Table 1.

**Table 1:** SEM Settings, EBSD and λCL collection and processing variables.

**SEM Settings**	***Technique***	**EBSD**	**Panchromatic CL**	**λCL**
SEM		Philips XL30	Philips XL30	Jeol JXA8200
Detector		Nordlys 1	CCD-Si (K.E. Developments Ltd)	2049-element linear spectrometer/CCD-Si
Analytical/acquisition system		HKL Channel 5	Philips	XCLent (CSIRO)
C coat		Y	Y	Y
Working distance (mm)		20	15	11
Tilt (°)		70	0	0
Accelerating voltage (kV)		20	10	20
Spot size (μm)		~0.5(#5)	~0.5(#7)	2
Probe current (nA)		N.M.	N.M.	100 nA

**EBSD Settings**			**λCL settings**	

EBSP collection time per frame (ms)		60	Sensitivity (nphotons/count)	86
Background (frames)		64	Spectral range (nm)	331.4 to 1826.9
EBSP noise reduction (frames/binning)		4/4 × 4	Dwell time per point (ms)	50
Step size (μm)		5	Grating (lines/mm)	300
***Indexing settings***			Blaze width (nm)	500
Reflector file		Card 5260*	Step size (μm)	2
Band detection (*n *bands)		8	Grid	250 × 250
Hough resolution		60		
Average mean angular deviation (°)		0.3223		
***Noise reduction***				
Wildspike (% of total data)		0.0002		
6 neighbor zero solution extrapolation (% of total data)		0.85		
Orientation averaging filter** (Filter size/smoothing angle/artifact angle)		3 × 3/5°/1°		

N.M. = not measured. * = generated from Card No 5260 of the Mincryst crystallographic and crystallochemical database [38]. ** = detailed in Humphreys et al. [39]

Full crystallographic orientation information was obtained by collection of electron backscatter patterns (EBSPs) at individual points which were indexed by fitting a solution generated from a reflector file for zircon (Table 1) [38]. EBSD maps were acquired by automated collection of 5 μm spaced nodes on a 100 × 100 grid. Data were collected from 64 individual maps that were stitched to provide a single map of the whole grain. The angular fit between the EBSP at each point and the theoretical index solution, given by the mean angular deviation (MAD), was generally good, and all points are within the 1.3° indexing tolerance value, with a mean MAD value for the stitched map of 0.3223°. Orientation data from individual maps were collected prior to the development of a dynamic adjustment of projection parameters which corrects for angular variations in the beam-sample geometry during scanning. Consequently, the stitched map contains artifact misorientations at map joins resulting from a cumulative systematic orientation variation of ~1.4° over the area of individual maps.

The EBSD data was noise reduced using the 'Tango' module of HKL Technology's Channel 5.0.9.0^© ^software. A 'wildspike' correction was applied to remove isolated misindexed points, and individual analyses that had no solution were infilled by a 6 neighbor extrapolation (<1% of total data, Table 1). An additional orientation averaging filter [39] was also applied to reduce the noise associated with 0.5–2° boundaries (Table 1). Comparison of the data before and after this noise reduction procedure indicates no artifact generation.

Where adjacent pixels are differently oriented then their orientation relationship can be expressed as an angular rotation about an axis, referred to as misorientation. For misorientation analysis, the minimum misorientation (also known as disorientation) angle/axis pairs were calculated for adjacent pixels. Cumulative orientation maps were produced using the Channel 5 'texture' component, in which each pixel was colored for minimum misorientation relative to a user-defined orientation. The local misorientation map involved calculation of the mean of the minimum misorientation values between a central pixel and its first and second nearest neighbors in a 5 × 5 filter grid. Artifact misorientations at stitched map joins were omitted from misorientation analysis.

Isotopic data were collected using the SHRIMP II at the John De Laeter Centre, Curtin University of Technology, Perth, using well established procedures [40]. Primary beam current was 3nA, and mass resolution was 5000. Isotope ratios and elemental concentrations were calibrated using the Curtin University standard CZ3 (564 Ma; ^206^Pb/^238^U = 0.0914, 550 ppm U), and data were reduced using Krill software (developed by P. Kinny). Correction of Pb isotope ratios for common Pb was based on the measured ^204^Pb, representing up to 4% correction to the measured ^206^Pb. Uncertainty in element concentration is estimated at ± 20% based on the reproducibility of analyses of CZ3 which has a uniform U content of 550 ppm. SHRIMP analysis points were carefully positioned to avoid surface topographic features such as fractures and fluid inclusion pits, and were located with reference to orientation maps from EBSD data.

Panchromatic cathodoluminescence (CL) imaging was done at Curtin University of Technology, Perth (Table 1). Relative panchromatic CL intensities were calculated using the image processing software Scion Image^© ^Beta 4.0.2 (Scion Corporation, 2000). CL imaging was done after geochemical analysis to accurately determine the position of SHRIMP analysis spots. Wavelength CL (λCL) data was collected at the Advanced Analytical Centre, James Cook University, Townsville, Australia. System and setting details are given in Table 1. A 500 × 500 μm area was mapped with a grid spacing of 2 μm, and the data was managed through XCLent operating software [41].

Two approaches were used to facilitate quantitative correlation between deformation microstructure and geochemistry. The first involved comparison between the cumulative misorientation values at the centre of the SHRIMP spot with element abundance, which allowed correlation of geochemical variations with the long-range lattice distortion. The second approach involved correlation of the local misorientation value of the pixel in the centre of the SHRIMP spot with geochemistry, and tested the effect of short-range or 'local' misorientation on geochemistry. The advantage of the latter approach is that the local misorientation value accounts for the microstructural heterogeneities in a 25 × 25 μm area, which is broadly at the same scale as a SHRIMP analysis spot.

## Results

### EBSD data

**Figure 2:**
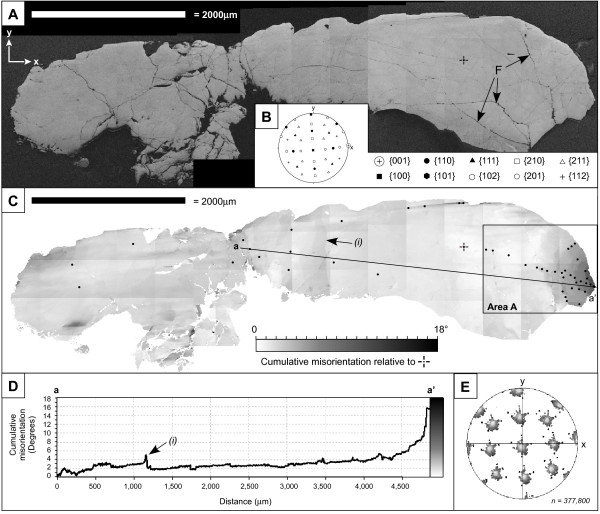
Maps of the whole zircon grain derived from EBSD data. Maps contain artifacts resulting from stitching several smaller maps. a) Band contrast map where pixel values correspond to the contrast in hough space, and is a measure of pattern quality. This map shows the external morphology of the grain position of intragrain fractures (e.g., F). The x,y indicates the arbitrary sample reference frame used throughout the study. b) Lower hemisphere equal area stereographic projection of the poles to low-index planes for the reference orientation shown by a cross in (a). Data plotted in the sample reference frame. c) Cumulative misorientation map in which each pixel is coloured for minimum misorientation relative to the reference orientation shown in (a) and (b). This shows lattice distortion is predominantly localised at the grain tips. The right hand box defines a domain (area a) of cumulative misorientation up to 18°, and is the focus of this study. (i) Shows a cross-cutting linear feature that defines an abrupt change in orientation. d) Cumulative misorientation profile for line a-a' shown in (b). The position of a cross-cutting linear feature (i) is indicated by an arrow. e) Lower Hemisphere equal area stereographic projection of showing the dispersion of poles to low-index planes across the whole grain.

The zircon grain is orientated such that the c-axis of the grain is broadly parallel with the sample surface, and approximately lies in the x direction (in the user-defined, arbitrary sample coordinate system), while the symmetrically equivalent a-directions closely correspond to y and z (Fig. 2a,b). Multiple sets of broadly-spaced brittle fractures cut across the grain, and are particularly prevalent in the left-hand side part of the grain (Fig. 2a). EBSD mapping reveals that the grain contains variations in crystallographic orientation (Fig. 2c–e). The central part of the grain contains broad areas with relatively consistent orientation separated by a small number of discrete linear zones that transect the short axis of the grain and which accommodate 1–5° misorientations (Fig. 2c,d). The distribution pattern of crystallographic poles indicates a smooth and continuous dispersion in multiple directions from the reference orientation (Fig. 2e).

**Figure 3:**
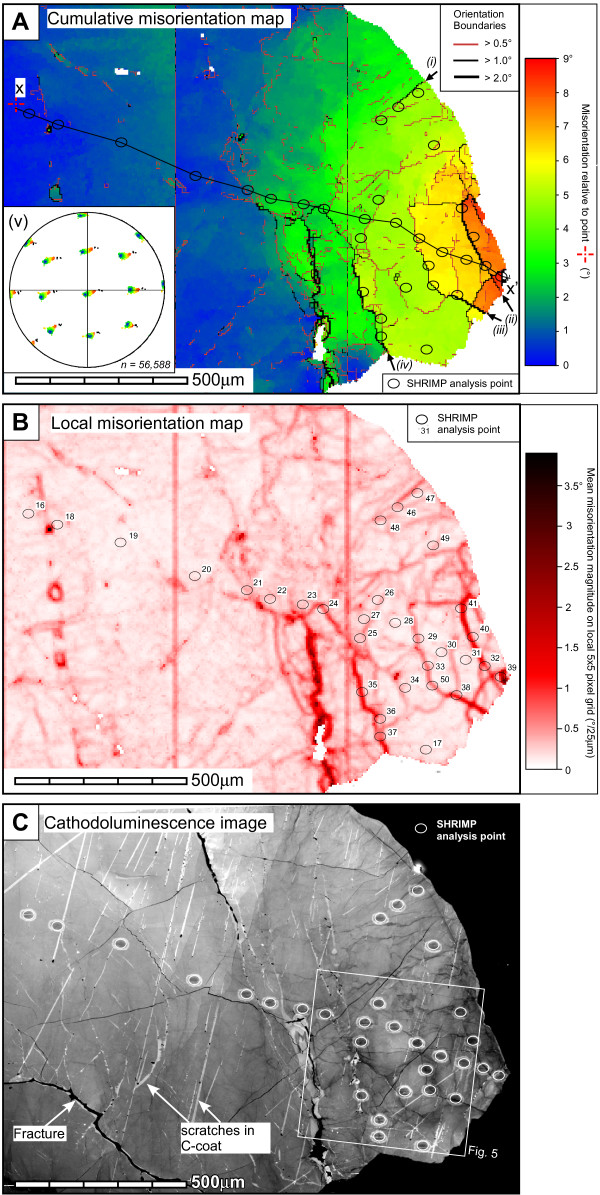
Maps of area A shown in Fig. 2c. a) Cumulative misorientation map derived from EBSD data showing disorientation relative to reference orientation shown by a cross. Boundaries between adjacent pixels with disorientation angles of >0.5°, >1°, and >2° are shown as solid lines. Misorientation axes for boundaries (i) to (iv) are shown in Fig. 5. (v) Stereographic projection of poles to low index crystallographic planes for all pixels indexed as zircon in shown in (a). b) Local misorientation map derived from EBSD data. Each pixel is coloured according to the mean disorientation value from a surrounding 5 × 5 pixel grid. Misorientations that define coarse vertical grid lines in (a) and (b) are an artefact of data collection (see text), and were disregarded for misorientation analysis. The position of SHRIMP analyses are indicated on each map. Profile along line x-x' is shown in Fig. 6. c) Panchromatic cathodoluminescence (CL) image. The locations of SHRIMP analyses are shown. Pale linear features are scratches in the C-coat.

Much of the orientation variation occurs in the right-hand side tip of the grain which shows a gradual cumulative misorientation of over 12° over a distance of ~700 μm (Fig. 2c,d). In this tip, the gradual orientation gradient is punctuated by discrete domain boundaries that accommodate <5° misorientations – low-angle boundaries – that do not relate to data acquisition artifacts (Fig. 3a). Low-angle boundaries increase in abundance toward the tip and define an interconnected network and separate domains of relatively consistent internal orientation (Fig. 3a,b). The misorientation magnitude of individual low-angle boundaries varies along their length, particularly at sharp bends, or at intersections with other boundaries. The low-angle boundary network does not have a surface topographic expression, and is cut by, and therefore predates visible brittle fractures. The misorientation across late brittle fractures is minimal (<0.5°), and the cumulative misorientation across all fractures in the tip shown in Fig. 3 does not exceed 1°.

**Figure 4:**
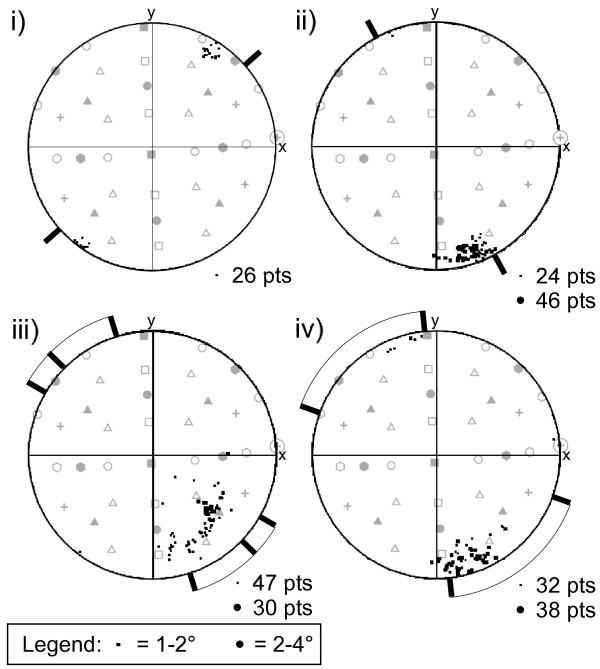
Lower hemisphere equal area stereographic projections of disorientation axes along low-angle boundaries (i) to (iv) indicated on Fig. 3a. Grey symbols are low index poles of the reference orientation 'x' in Fig. 2b. Refer to Fig. 2b for legend. Thick black lines extending outside the primitive circle indicate the range of boundary trace orientations on the sample surface.

The dispersion pattern of the main crystallographic poles for all data from the tip is complex, and indicates that the crystallographic pole data are rotated around more than one axis (fig. 3a.v). The dispersion pattern is dominated by rotation about an axis that plunges shallowly to the SE which is a similar trend to most of the >1° boundaries (Fig. 3a,b). The misorientation axes associated with each >1° boundary cluster in consistent orientations and lie at a low angle to the trace of the boundary, but do not necessarily coincide with low index directions for zircon (Fig. 4).

### Cathodoluminescence analysis

**Figure 5:**
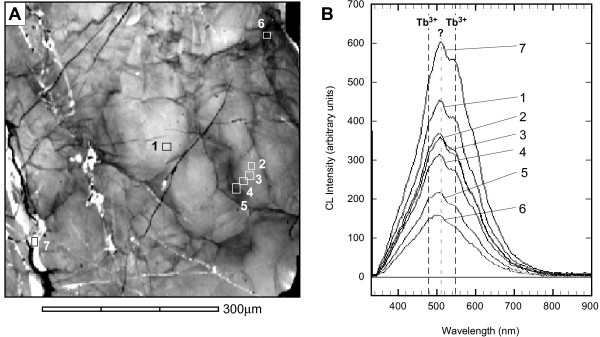
(a) Wavelength CL map of detailed area shown in Fig. 3c, shaded for integrated CL intensity. (b) CL spectra for points 1 to 8 shown in (a). Each spectrum represents mean values from a 3 × 3 pixel local grid. A broad double peak at ~500nm and ~540nm is common to all spectra. See text for discussion.

The grain centre shows little variation in CL intensity and a broad, gradual decrease in CL signal toward the grain tip. The only concentric zoning as shown by CL imaging is a narrow (<20 μm), discontinuous rim of low CL intensity (Fig. 3c). Late brittle fractures appear as distinctive discrete non-luminescent bands. A complex network of locally CL-dark domains occurs at the grain tip (Fig. 3c, 5a). These linear features do not have sharp boundaries (as expected if they were brittle fractures), they do not relate to topographic features on the sample surface, and are cross-cut by (and so pre-date) all sets of late brittle fractures (Figs 3c, 5a). Instead, CL intensity reduction into and along the length of interconnecting networks of 3–15 μm wide linear zones is gradational with locally varying gradients. In some instances, narrow zones with steep CL gradients terminate into broader domains of little CL reduction (Figs 3c, 5a). The λCL spectrum in a CL-bright subgrain interior shows a broad emission band between ~340 and ~820 nm, centred at ~510 nm with two minor peaks at ~510 nm and minor peaks at ~490 and ~550 nm (Fig. 5b). Analysis λCL spectra at points along a transect from CL-bright subgrain into CL-dark low-angle boundary domain shows a progressive reduction in the integrated CL intensity with no shifts in the peak positions or relative heights/peak shape. The right-hand tip also contains localized and discontinuous narrow linear patches of bright CL zircon with sharp boundaries that crosscut the low-angle boundary microstucture. These features have the same characteristic λCL emission spectral peak shape and position as the host zircon, but have significantly elevated relative intensity (Fig. 5). These microstructures either represent fractures filled with new zircon (i.e. zircon veins), or domains of solid-state recrystallization of the host zircon, perhaps localized along pre-existing, late brittle fractures.

### Geochemical analyses

**Figure 6:**
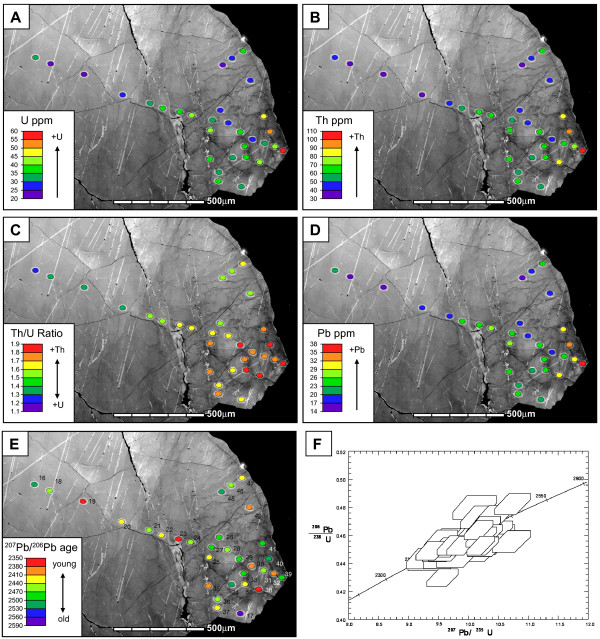
(a) to (e) Maps to show spatial variations in trace element geochemistry and isotope data. a) U concentration.(b) Th concentration. c) Th/U ratio. d) Pb concentration. e) ^207^Pb/^206^Pb age. Base image is panchromatic cathodoluminescence image shown in Fig. 3a. (f) Concordia plot of all U-Pb SHRIMP analyses for whole grain (n = 50). Error boxes are 1σ. All data are ≥ 94% concordant. The combined mean ^207^Pb/^206^Pb age is 2,451 ± 14 Ma, with an MSWD of 1.3.

**Figure 7:**
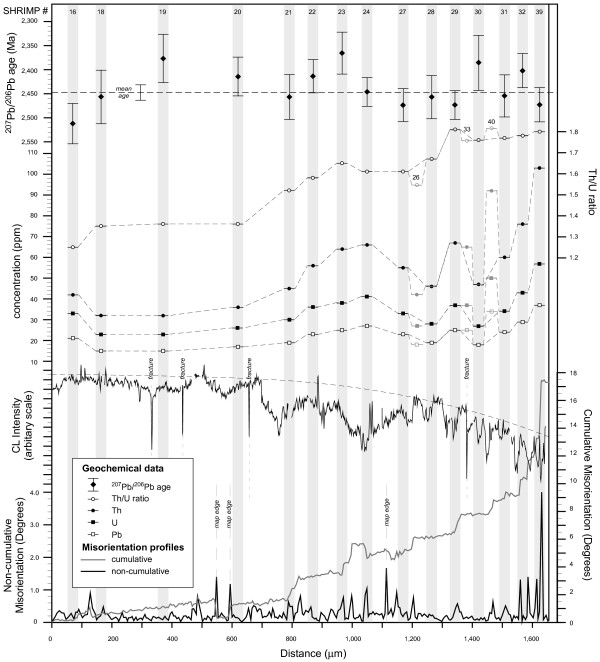
Profile of misorientation, panchromatic CL intensity, and geochemical data along line x-x' shown in Fig 3. Vertical grey bars represent areas covered by SHRIMP analyses. Errors in ^207^Pb/^206^Pb ages are 1σ. Mean ^207^Pb/^206^Pb age is for the whole grain (n = 50). Trace element concentrations for SHRIMP analyses are given nominal ±20% errors. Grey geochemistry symbols show values for analyses immediately adjacent line x-x'. Sharp reductions in CL intensity associated with brittle fractures are shown. Artefact misorientations at map edges are shown. Dashed lines are to guide the eyes only. See text for further interpretation.

Analytical results of thirty four new SHRIMP analyses across the grain are listed in Table 2. Geochemical analysis points were positioned such that a variety of differently-oriented low-angle boundaries of different misorientation magnitudes, orientation domain interiors and undeformed central parts of the grain were sampled (Fig. 3). Changes in U and Th are broadly covariant, but in accordance with the absence of visible growth zoning under CL the overall distribution of trace elements in the grain does not appear to follow any regular concentric or radial pattern of the kind that is usual for igneous zircon. It is noticeable, however, that the deformed part of the grain contains much more variable U and Th values than the undeformed zircon (Figs 6a,b and 7). Fifteen of the thirty-four analyses collected for this study define a transect from the undeformed to the deformed part of the grain (Figs 3a and 7). The grain contains variations in U from 20–60 ppm and Th from 30–110 ppm.

**Table 2:** SHRIMP U-Pb data for the zircon.

Spot	U (ppm)	Th (ppm)	Th/U	Pb (ppm)	c^206^Pb (%)	^207^Pb/^206^Pb	±1σ error	^206^Pb/^238^U	±1σ error	^207^Pb/^235^U	±1σ error	^207^Pb/^206^Pb age	±1σ error
16	33	42	1.25	21	1.47	0.1653	0.0041	0.4530	0.0080	10.33	0.33	2511	42
17	30	51	1.69	20	1.16	0.1708	0.0039	0.4557	0.0076	10.73	0.32	2565	38
18	23	31	1.35	15	2.21	0.1601	0.0053	0.4616	0.0082	10.19	0.41	2457	56
19	23	31	1.36	15	2.80	0.1528	0.0045	0.4799	0.0084	10.11	0.37	2378	51
20	26	36	1.36	17	1.91	0.1562	0.0037	0.4730	0.0079	10.19	0.31	2415	41
21	30	45	1.52	19	1.30	0.1602	0.0044	0.4442	0.0073	9.81	0.33	2457	47
22	36	56	1.58	23	1.60	0.1561	0.0032	0.4528	0.0069	9.75	0.26	2414	35
23	38	64	1.65	25	1.81	0.1518	0.0035	0.4562	0.0074	9.55	0.28	2366	39
24	41	66	1.61	27	1.37	0.1592	0.0028	0.4638	0.0068	10.18	0.25	2447	30
25	40	69	1.72	26	1.51	0.1585	0.0030	0.4527	0.0066	9.89	0.25	2439	32
26	27	42	1.55	18	2.00	0.1632	0.0044	0.4626	0.0077	10.41	0.35	2489	45
27	33	55	1.63	23	1.42	0.1616	0.0034	0.4824	0.0077	10.75	0.30	2473	35
28	28	46	1.67	19	2.28	0.1601	0.0043	0.4596	0.0077	10.14	0.34	2457	45
29	37	67	1.81	25	1.18	0.1616	0.0029	0.4588	0.0071	10.23	0.26	2473	30
30	27	47	1.76	18	3.73	0.1536	0.0053	0.4427	0.0077	9.37	0.38	2386	58
31	34	60	1.77	24	2.56	0.1597	0.0037	0.4737	0.0076	10.43	0.31	2452	39
32	43	76	1.78	29	2.24	0.1549	0.0033	0.4515	0.0068	9.64	0.27	2401	36
33	37	65	1.76	25	2.08	0.1621	0.0034	0.4607	0.0071	10.30	0.29	2478	36
34	30	51	1.68	20	1.90	0.1653	0.0046	0.4538	0.0076	10.35	0.35	2511	47
35	37	65	1.73	25	2.90	0.1544	0.0040	0.4484	0.0070	9.54	0.30	2395	44
36	32	53	1.66	22	2.97	0.1587	0.0045	0.4634	0.0075	10.14	0.35	2442	48
37	35	60	1.70	23	2.67	0.1567	0.0045	0.4445	0.0081	9.60	0.35	2420	49
38	44	80	1.82	30	2.64	0.1516	0.0032	0.4490	0.0067	9.39	0.26	2364	36
39	57	103	1.80	37	2.58	0.1616	0.0034	0.4296	0.0063	9.57	0.26	2473	36
40	50	92	1.83	34	1.51	0.1647	0.0028	0.4524	0.0065	10.27	0.24	2504	29
41	45	81	1.79	30	0.78	0.1636	0.0026	0.4527	0.0067	10.21	0.23	2494	27
42	25	34	1.37	16	2.28	0.1617	0.0072	0.4497	0.0084	10.03	0.50	2474	75
43	30	42	1.41	19	3.08	0.1525	0.0050	0.4434	0.0074	9.32	0.36	2374	55
44	37	48	1.29	24	1.84	0.1597	0.0034	0.4660	0.0071	10.26	0.29	2453	36
45	31	35	1.13	19	2.27	0.1594	0.0041	0.4571	0.0078	10.04	0.33	2449	44
46	26	40	1.53	18	2.99	0.1599	0.0053	0.4625	0.0081	10.20	0.40	2455	56
47	37	60	1.62	24	2.51	0.1557	0.0038	0.4487	0.0071	9.63	0.29	2410	41
48	24	35	1.51	16	1.94	0.1644	0.0047	0.4594	0.0081	10.41	0.37	2502	48
49	27	42	1.53	18	3.66	0.1545	0.0052	0.4568	0.0080	9.73	0.39	2396	58
50	35	63	1.80	25	2.83	0.1567	0.0041	0.4735	0.0076	10.23	0.33	2421	44

Ratios refer to radiogenic Pb, c^206^Pb is the percentage of common ^206^Pb in the total, as estimated from the measured ^204^Pb/^206^Pb ratio. The numeric suffix of each analysis corresponds to the numbered spots shown in Fig. 3c.

Total Pb concentration varies from 14–36 ppm among individual analyses, and its distribution follows that of U and Th concentration (Figs 6d and 7). There is very little variation in ^207^Pb/^206^Pb ages across the grain (Fig. 6e,f). All fifty analyses (34 from this study plus 16 from Kinny and Friend [34] combine to give a mean ^207^Pb/^206^Pb age of 2451 ± 14 Ma with an MSWD of 1.3 (Fig. 6f). The comparatively low MSWD indicates no significant excess scatter in the ^207^Pb/^206^Pb ratios beyond what is expected from their individually assigned analytical uncertainties, and the data can be interpreted as a single-age population. All but three of the analyses are over 95% concordant. Importantly, there is no discernible change in ^207^Pb/^206^Pb ages from core to rim, or any systematic change in apparent age or discordance with the deformation microstructure.

## Discussion

### Microstructural characterization and development

The gradual cumulative misorientation and the hierarchical network of low-angle boundaries typify microstructures produced by progressive crystal-plastic deformation processes (i.e., involving formation and migration of dislocations), rather than by solid-state recrystallization, dissolution/re-precipitation, or brittle fracture processes [22,42,43]. The gradual lattice distortions are accommodated by the formation of dislocations. Low-angle boundaries are consistent with formation by the accumulation of dislocations into high dislocation density walls that separate relatively dislocation free cells, or 'subgrains' and thus lowering the energy of the system. This process must have occurred at elevated temperatures where dislocation migration (glide) was enabled.

In certain circumstances, the misorientation axis geometry across low-angle boundaries can used to determine the causative slip system(s) in zircon [29]. The misorientation axes for individual low-angle boundaries vary and do not coincide with rational low-index directions, and represent the product of several combined operative slip systems. The position of the prevailing misorientation axes close to the pole to {100} suggests that there could be a dominant contribution of slip by either (001)<100> or (100)<001>, both of which are known slip systems in zircon [29]. However, explanatory solutions for mixed slip-systems are non-unique at the scale of the EBSD data, which prohibits the clear identification of causative slip systems and their relative contribution.

### Cause of the cathodoluminescence

The pattern of reduced CL intensity mimics the gradual cumulative misorientation and the spatial distribution of low-angle boundaries, and the steepest CL gradients correspond to boundaries with the highest misorientation (Fig. 3). This relationship is interpreted as a result of secondary modification of a CL-homogenous zircon. The effects of crystallographic orientation on CL cannot be ruled out for the broad trend of progressive CL reduction into the cumulatively misoriented grain tip (~12°). However, this cannot explain the intricate network of CL-dark domains (Figs 3, 5). The close correspondence of low-luminosity domains within and around low-angle boundaries would lead to lower general structural integrity (i.e., elevated dislocation density), and disruption of REE site symmetry, all of which have been proposed to significantly affect luminescence in zircon [44-46]. The broad peak shape of λCL spectra represents a composite envelope consisting of narrow REE emission multiplets superimposed on a broader emission peak. The two minor peaks at ~490 nm and ~550 nm correspond to Tb^3+ ^(489 nm and 548 nm), and possibly Er^3+ ^or Dy^3+^, which have overlapping peaks at 474 nm and 483 nm respectively [47]. The consistency of relative spectral peak heights into the deformed regions discounts relative changes in CL-active REE (e.g., Reddy et al. [28]), although the absolute intensity of REE bands is not necessarily linked to concentration [48]. Similarly, the maximum dose from α-decay associated with locally elevated U-Th content in deformed domains, even in the domain with the most elevated Th and U concentrations (6.48 × 10^8 ^events/mg [49]), is insufficient to cause radiation damage to suppress CL [46]. The strong, broad λCL peak centred at ~510 nm does not correspond with emission bands from known CL-active REE^3+^, or other known sources of luminescence in zircon [45,50-54]. The 510 nm peak is interpreted to be a consequence of dislocation-related defects. The strong and unambiguous response of total CL intensity to deformation presented here contrasts with the subtle effects of deformation-related homogenization of primary oscillatory CL zoning presented by Reddy et al. [28].

### Relationship between U, Th and Pb geochemistry and deformation microstructure

**Figure 8:**
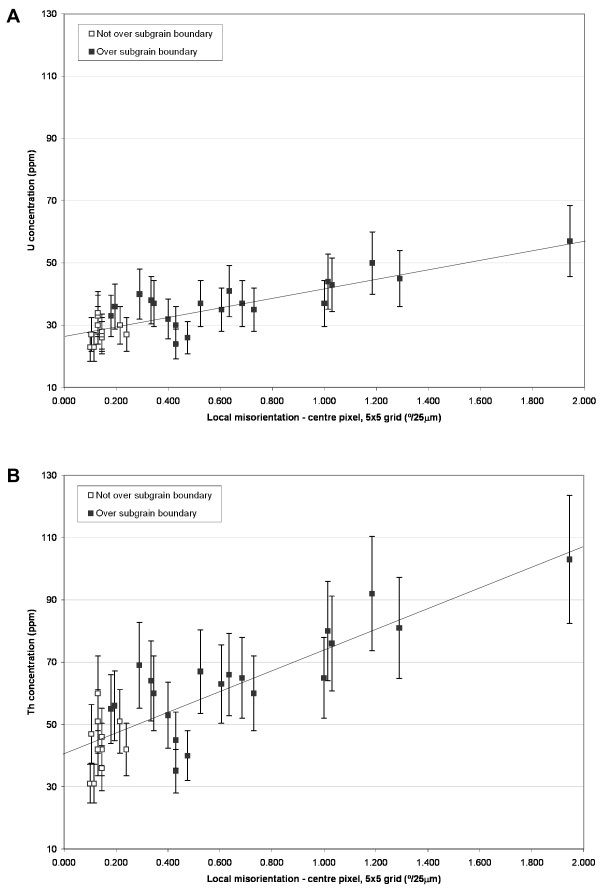
Plots of local misorientation versus (a) U and (b) Th concentration. Open boxes show analyses that do not lie over low-angle boundaries, closed boxes analyses over low-angle boundaries. Nominal ± 20% error bars are assigned to trace element concentration measurements. Data has been fitted with linear trend lines. See text for discussion.

The deformed grain tip contains elevated Th and U concentrations compared to the undeformed grain centre (Figs 2, 3, 6 and 7). However, superimposed on this trend are increased U and Th concentrations at low-angle boundaries (Figs 6a, 7, and 8) with the highest measured U and Th concentrations (spot 39, Figs 6 and 7) corresponding to the boundary that accommodates the largest misorientation (4°). The increase in U and Th concentration correlates with the magnitude of the mean local misorientation in the area of the SHRIMP analysis pit (Fig. 8). It should be noted that low-angle boundary widths are a fraction of the diameter of SHRIMP pits (Figs 3, 6 and 7), and the actual U-Th enrichment in low-angle boundary domains is more extreme than the data shows due to the homogenization effect of analytical volumes during SHRIMP analysis.

This correlation indicates that the U and Th levels are related to deformation microstructures, and therefore variations are not consistent with volume diffusion. Several studies have documented that the bulk diffusion properties of deformed materials can be enhanced by several orders of magnitude over volume diffusion in a pristine lattice [28,31,55-57]. Individual dislocations, and low- and high-angle boundaries are all high-diffusivity pathways, and their development can lead to complex multipath diffusion [29,57-60]. These pathways can be static, and/or migrate through the grain and be transient (especially for glissile individual dislocations), allowing interaction with volumes of subgrain interiors. Stress fields that surround dislocations can also facilitate diffusion of trace element impurities into their cores – so called Cottrell atmospheres [61-63] – which leads to enrichment in domains with high dislocation density (e.g., at low-angle boundaries).

### Timing of deformation

The distribution of Pb mimics that of U and Th. Individual SHRIMP analyses are highly concordant, with no systematic variation in ^207^Pb/^206^Pb ages with deformation. An interpretation that deformation only affects U and Th but not Pb mobility goes against experimental and theoretical studies which predict that diffusivity of Pb is far greater than U and Th by orders of magnitude [13-15]. Movement of Pb decoupled from U and Th would be expressed as sites of normal (Pb deficient) and reverse (Pb enriched) discordance, but this was not found. A more satisfactory explanation is that the grain was deformed shortly after initial crystallization, and that the exact timing of any deformation-related isotopic resetting is not resolvable using the available techniques. The combined ^207^Pb/^206^Pb age of 2451 ± 14 Ma indicates that the grain post-dates Badcallian peak metamorphism (2,480–2,490 Ma) by at least 16 Ma, pre-dates the Laxfordian event, and that zircon growth and deformation probably occurred during Inverian metamorphism at amphibolite facies conditions. The data show that no significant microstructure-related isotopic disturbance (e.g., Pb-loss) has occurred subsequently at lower-grade conditions, long after the deformation microstructure was established. In addition, the U-Th-Pb systematics indicate U-Th mobility occurred either entirely during the dynamic deformation processes, or immediately after deformation at high temperatures.

### Sources of U and Th

In addition to the increase in U and Th there is a relative increase in Th over U in the deformed zones, which translates to a systematic increase in the Th/U ratio from 1.13 to 1.83 (Figs 6c and 7). Th/U ratio is highest in areas of high local misorientation (i.e., over low-angle boundaries) (see Fig. 8). Data that plot off the linear trend could reflect slight variations in pre-deformation U and Th concentration. The localized increase of U and Th could be due to their internal redistribution, or be derived from a source external to the grain. If the trace element distribution is purely a result of internal redistribution during deformation by Cottrell processes and/or carrying of elements by the migration of individual dislocations, then volumes of material that have been swept by dislocations or adjacent low-angle boundaries should be depleted in trace elements [64], which is not shown by the geochemical data. It also seems unfeasible that dislocations have swept the whole grain to eventually rest at the grain tip. A more realistic explanation is that deformation commenced at or near the grain tip due to a locally-elevated stress field (impingement with other grains?), and progressively 'migrated' into the grain as it accommodated higher strain. The bulk U and Th enrichment in all deformed areas and absence of trace element depleted zones relative to undeformed zircon imply a net influx of U and Th to the grain from an external source, such as a U-Th enriched grain boundary fluid phase.

A chemical gradient between zircon and a source highly enriched in incompatible U and Th could provide a driving force for trace element diffusion into zircon. The systematic increase in Th/U with misorientation implies enrichment in Th relative to U in response to deformation that reflects either a difference in element mobility or a high Th/U ratio source. The greater enrichment in Th may result from the diffusivity of Th being greater than U. However, this is inconsistent with the slightly enhanced 'volume diffusivity' of U^4+ ^over Th^4+ ^within zircon, which is thought to relate to differences in ionic radii of ~1.05 Å and ~1.10 Å in eight-fold coordination, respectively [9,12]. Alternatively, the elevated Th/U ratio in the deformed domains reflects a high Th/U external source. U and Th could be supplied via grain boundary diffusion of ions liberated by resorption of nearby U-Th-bearing phases, such as monazite. However, the high Th/U ratios of modified zircon (up to 1.83) are uncharacteristic of zircon grown during high grade metamorphism, which typically has Th/U ratios <0.07 [2,10]. Alternatively, the substantial increase in Th/U ratio with deformation reflects deformation of the grain in the presence of a grain boundary fluid phase with high Th/U ratio, possibly a late stage evolved magmatic fluid associated with the host pyroxenite pegmatite. This is supported by the ^207^Pb/^206^Pb data that suggests that deformation occurred shortly after initial crystallization.

### Implications

Electron backscatter diffraction has been successfully used to identify deformation microstructures and explain variations in U-Th in zircon, and preliminary analyses of zircon from a range of different geological environments shows that crystal-plastic deformation in zircon may be quite common (Timms and Reddy, unpublished data). The present study and other studies show that zircon can deform by crystal-plasticity at amphibolite facies conditions [28,29]. However, the full range of conditions over which zircon is able to deform by crystal-plastic processes has not yet been constrained, and currently there are no published studies that systematically test the extent to which a given population of zircon grains can be affected by rock deformation. The relative importance of numerous interacting factors such as, temperature, pressure, presence of fluids, total bulk strain, strain partitioning, and the relative strength of other mineral species in the host rock, as well as the microstructural setting of the zircon grains, are yet to be demonstrated. This research highlights the need to study other examples of zircon with protracted deformation histories.

The particular circumstances of the zircon grain in this study have resulted in modification of the U-Th-Pb system without generation of discernable variations in ^207^Pb/^206^Pb ages. However, in situations where high-temperature deformation occurs at a much later date than zircon crystallization, there could be dramatic variations in the U-Th-Pb compositions and ^207^Pb/^206^Pb ages. The differential enrichment of Th over U illustrates that deformation-enhanced diffusion could potentially result in the significant modification of Th/U ratio, and implies that Th/U ratio may not always be a robust indicator of primary crystallization environment. The possibility that the trace element geochemistry can be modified by processes associated with deformation has important implications for all applications of zircon research, yet intragrain plastic deformation of zircon has generally been overlooked.

Deformation microstructures (commonly <10° orientation variations) may be masked in optical microscopy by zircon's high birefringence, especially given the small sizes of grains common to most rock types. The effects of deformation on CL images can be very subtle and could go unnoticed [28]. We have demonstrated in this study that intragrain deformation microstructures with angular changes of only a few degrees can be important for U-Th mobility in zircon, a finding in agreement with REE mobility [28]. Detection and characterization of these subtle microstructures at the grain scale by EBSD mapping was paramount in both studies. The spatial scale of deformation microstructures in zircon associated with chemical mobility are much finer than the spatial resolution of current proton-, ion- and laser-probes and instruments popular for in-situ geochronology/geochemistry studies, and can affect significant areas of grains. Therefore, potentially erroneous interpretations of geochemical data could result without at least first identifying and characterizing deformation microstructures.
